# Leveraging word embeddings and medical entity extraction for biomedical dataset retrieval using unstructured texts

**DOI:** 10.1093/database/bax091

**Published:** 2017-12-20

**Authors:** Yanshan Wang, Majid Rastegar-Mojarad, Ravikumar Komandur-Elayavilli, Hongfang Liu

**Affiliations:** Department of Health Sciences Research, Mayo Clinic, Rochester, MN 55901, USA

## Abstract

The recent movement towards open data in the biomedical domain has generated a large number of datasets that are publicly accessible. The Big Data to Knowledge data indexing project, biomedical and healthCAre Data Discovery Index Ecosystem (bioCADDIE), has gathered these datasets in a one-stop portal aiming at facilitating their reuse for accelerating scientific advances. However, as the number of biomedical datasets stored and indexed increases, it becomes more and more challenging to retrieve the relevant datasets according to researchers’ queries. In this article, we propose an information retrieval (IR) system to tackle this problem and implement it for the bioCADDIE Dataset Retrieval Challenge. The system leverages the unstructured texts of each dataset including the title and description for the dataset, and utilizes a state-of-the-art IR model, medical named entity extraction techniques, query expansion with deep learning-based word embeddings and a re-ranking strategy to enhance the retrieval performance. In empirical experiments, we compared the proposed system with 11 baseline systems using the bioCADDIE Dataset Retrieval Challenge datasets. The experimental results show that the proposed system outperforms other systems in terms of inference Average Precision and inference normalized Discounted Cumulative Gain, implying that the proposed system is a viable option for biomedical dataset retrieval.

**Database URL**: https://github.com/yanshanwang/biocaddie2016mayodata

## Introduction

The recent movement towards open data in the biomedical domain has generated a large number of datasets that are publicly accessible (1–3). It not only makes research transparent and reproducible, but also allows for more collaborative and rapid progress and enables the development of new questions by revealing previously hidden patterns and connections across datasets (4). Due to the lack of standards, however, integration and interconnection of datasets available in different repositories are major obstacles for biomedical research (5, 6).

There have been considerable efforts that attempt to address the integration issue. For example, a number of scientific journals have created policies about sharing data. Many projects have been funded to tackle the biomedical data integration problem, such as the OpenAIRE project (http://www.openaire.eu/) in Europe and the Open Research Data project (http://www.rcuk.ac.uk/research/opendata/) in UK. In the US, the National Institutes of Health has funded the biomedical and healthCAre Data Discovery Index Ecosystem (http://biocaddie.ucsd.edu/) (bioCADDIE) prototype through the Big Data to Knowledge program. The bioCADDIE is a data discovery index prototype providing a searchable index of biomedical study data, analogous to what PubMed and PubMed Central have achieved for medical literature (4, 7). However, as bioCADDIE has ingested and indexed >840 000 datasets from 23 different repositories across 10 different data types (8), it becomes more and more challenging to retrieve the datasets that meet the needs of the biomedical researchers.

With this in mind, the bioCADDIE Dataset Retrieval Challenge (9) was initiated with a goal of addressing the dearth of tools to retrieve relevant datasets from a large collection of biomedical datasets, in order to facilitate the re-utilization of collected data, and to enable the replication of published results. Specifically, the task is an information retrieval (IR) task that is defined as follows: Given a biomedical researcher’s query, participants were challenged to retrieve 1000 biomedical datasets relevant for answering a specific instantiated query. Retrieved datasets will be manually judged by human annotators and categoried into three levels of relevance, i.e. relevant, partially relevant or not relevant, according to whether or not they meet all the constraints specified in the query. Multiple metadata, including structured, unstructured and semi-structured metadata, were given in the dataset collection, such as ‘title,’ ‘description,’ ‘platform,’ ‘repository’ and ‘species.’

In this article, we describe an IR system for the bioCADDIE Dataset Retrieval Challenge and focus on using the unstructured textual data, specifically, ‘title’ and ‘description.’ The system utilizes a state-of-the-art IR model, medical named entity extraction techniques, query expansion with deep learning-based word embeddings and a re-ranking strategy to enhance the retrieval performance. In empirical experiments, we compared the proposed system with 11 baseline systems using the bioCADDIE Dataset Retrieval Challenge datasets.

The article is organized as follows. First, we briefly review related work. Second, we described the proposed methods, including the IR model, medical entity extraction, query expansion with word embeddings and the re-ranking mechanism. Third, we present the experiments including the data given in the challenge, preprocessing, indexing and experimental results. Finally, we conclude the article with discussions, limitations and future directions.

## Related work

**Figure 1.**
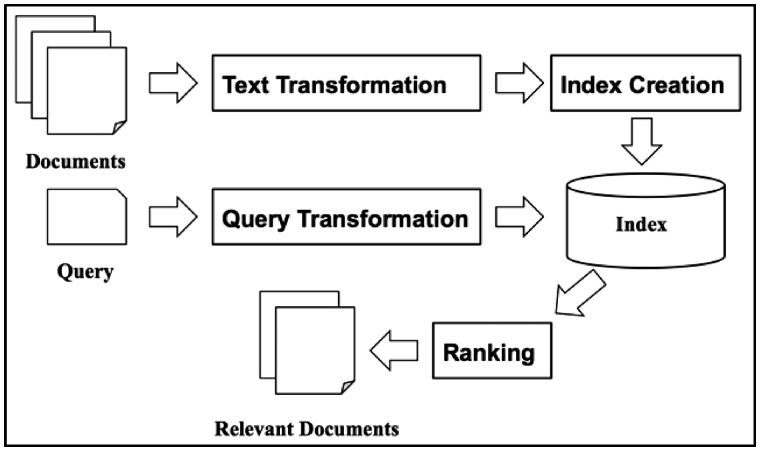
A basic IR architecture.

In this big data era, we always find it challenging to find the most relevant documents to a query from a large collection of documents. IR has been studied to address this issue for decades. IR techniques have been adopted in every search engine for searching the World Wide Web. A typical scenario is that a user inputs a query into a search engine and the search engine retrieves answers in the form of a list of documents in ranked order (10). According to the classic definition of IR in (11), ‘IR is a field concerned with the structure, analysis, organization, storage, searching and retrieval of information.’ Figure 1 shows a high-level IR architecture, which consists of two major functions, indexing and querying. The indexing process creates the structures that make document contents searchable while querying takes a user’s query as input and uses retrieval algorithms and those indexing structures to produce relevant documents in the order of ranking scores.

In the indexing process, text transformation and index creation are two major components. The conventional method of text transformation is to transform documents into index terms. An alternative is to use vectors for representing document contents. The vectors might refer to an index term or partial document. Vector Space Model (VSM) is the most widely used method in vector representations (12, 13). There are different variants of VSM based on how the vectors are generated. Tf-idf is the simplest method that calculates the term frequency-inverse document frequency for each term (12, 13). Latent Semantic Analysis (14, 15) and Latent Dirichlet Allocation (16, 17) are topic modeling methods that could capture some aspects of hidden conceptual information and represent such information in vectors.

**Figure 2.**
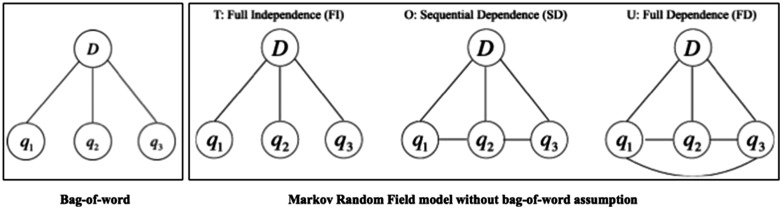
An example of bag-of-word assumption and MRF model.

Unlike the VSM model that assumes words are independent of each other (i.e. bag-of-word assumption), the Markov Random Field (MRF) model is a state-of-the-art IR model that leverages Markov properties to take into account the relationships between terms (18). Figure 2 illustrates an example of the bag-of-word assumption and the MRF model with three dependency types. The MRF model explicitly represents three types of dependencies between query terms. It has been verified on a variety of IR tasks and the performance has shown promise compared to the conventional bag-of-word based models (18, 19). Recently, Wang *et al.* proposed a Part-Of-Speech (POS) based MRF (POS-MRF) model, which is a variant of the MRF model that assigns different weights to different query terms according to the terms’ POS (20). It outperforms the conventional MRF model based on exhaustive experiments (20, 21). Therefore, we also utilized the POS-MRF in our proposed system.

In the querying process, query transformation and ranking are two major components. Query transformation is important for the final retrieval performance since a raw query might not fully capture the linguistic variability of the information needs. Query transformation includes simple stopwords removal, stemming and more sophisticated spell checking and query term suggestion. In addition, query expansion is a commonly adopted technique in query transformation that expands an initial query using synonyms and semantically related words (22, 23). However, it is still an open question how to find the most related words automatically. Some researchers use topic modeling to expand queries with terms having shared latent topics (24).

Recently, deep learning has drawn researchers’ interest since it automatically learns features from data. Word embeddings are one of the widely used word representations that are trained by deep learning models, which represent words in a dense low-dimension vector that captures hidden features of the word. Having been verified by many winning systems in the Text Retrieval Conference (TREC) Clinical Decision Support (CDS), word embeddings have been shown to be effective for query expansion. The most commonly used model for generating word embeddings is word2vec (25). Many participants in the TREC CDS 2016 (26) have used word2vec to expand queries with semantically related terms (27, 28). The difference between their methods is that distinct corpora were utilized to train the word2vec. Jo and Lee (27) and Gurulingappa *et al.* (29) used Wikipedia to train word embeddings while Greuter *et al.* used the TREC-supplied corpus. Diaz *et al.* (30) showed some substantial evidence that word embeddings trained on a global corpus, such as Wikipedia, under-performed those trained on local corpora for IR tasks, particularly for query expansion. Therefore, in our approach, we used word embeddings that were trained on the supplied corpus to expand queries.

Ranking is another crucial component in the querying process since it determines the position of a relevant document in the final retrieval list. A ranking algorithm is able to rank the relevant documents at the top of the list. Many ranking algorithms have been proposed in the literature, such as BM25 (31) and a query likelihood ranking model (32). It has been shown that the Dirichlet smoothing-based query likelihood model performs better than other models (33). Thus, it was used in the proposed system.

In the biomedical domain, IR tasks mainly focus on retrieving relevant biomedical literature to help physicians and clinicians make better decisions in patient care. The TREC CDS track is an IR shared task that aims to provide common biomedical datasets for participants and promote biomedical IR research (34). Most participants in the TREC CDS utilized medical knowledge to enhance their IR methods. The Unified Medical Language System (UMLS) was the most widely used medical knowledge base (35–37). Jo and Lee (27) utilized the UMLS to construct a clinical causal knowledge to re-rank retrieved documents. Other systems utilized UMLS to expand queries with its thesaurus (29, 38). In addition to the UMLS, Medical Subject Headings (MeSH) (39), Systematized Nomenclature of Medicine–Clinical Terms (SNOMED CT) and Wikipedia were also utilized as medical knowledge bases. Mourao *et al.* (40) appended synonyms, alternative and preferential labels for all query terms using SNOMED CT and MeSH. Nikolentzos *et al.* (41) expanded queries with extracted terms from Wikipedia. All these studies showed improvement over their baselines without using a medical knowledge base.

## Materials and methods

In this section, we present an overview of the proposed system and detail each component in the system.

### System overview

**Figure 3.**
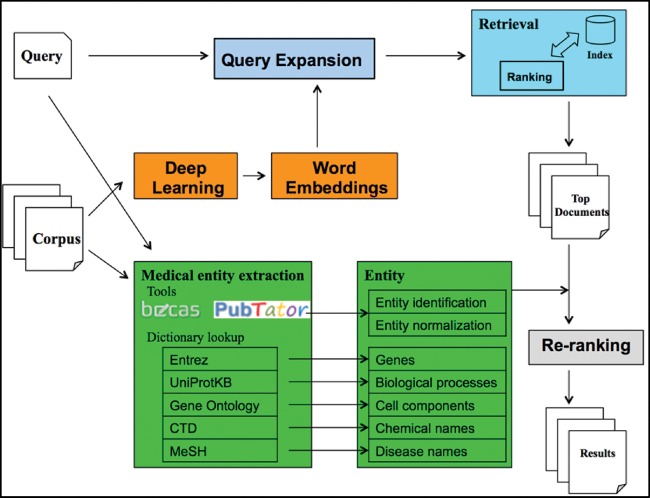
System overview of the proposed method.

Figure 3 depicts an overview of the proposed system. Overall, the system contains three parts: query expansion, IR model and re-ranking. We describe each step below.

Query expansion. We utilized the corpus containing all the unstructured texts (i.e. ‘title’ and ‘description’) of the datasets and trained the skip-gram model (25), a word2vec model, to obtain the word embeddings. Then, we expanded each medical term in a query with the five nearest terms in the embedding space.

IR model. We indexed the ‘title’ and ‘description’ from each dataset into two separate fields and utilized the POS-MRF model to query the two fields simultaneously to retrieve the relevant datasets. In this article, we also use *document* to represent the two fields of a specified dataset.

Re-ranking. An ensemble of state-of-the-art named entity recognition and normalization tools were applied to extract medical entities, such as genes and chemical names, from both corpus and queries. Then we re-ranked the top 10 000 retrieved datasets in the previous step by counting the shared entities between documents and queries. By doing so, the datasets that contained more identical medical entities were ranked higher in the final 1000 documents.

### Retrieval model

**Figure 4.**
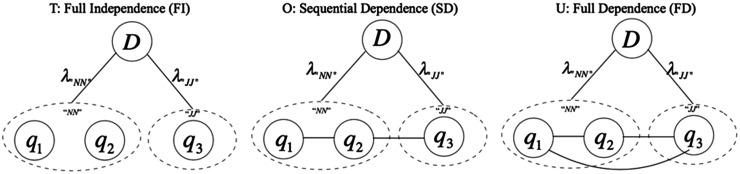
An example of the POS-MRF model.

POS-MRF is a variant of the MRF model that leverages the grammatical property POS to assign weights to different words (20). Figure 4 shows an example graphical model of the POS-MRF model with three query terms. Similar to the MRF model, the POS-MRF model contains three dependency types, namely full independence (denoted as *F*), sequential dependence (denoted as *O*) and full dependence (denoted as *U*). Alternatively, a term weight, denoted as $\lambda_{t}$, is assigned to each query term according to its POS category t. The joint probability function of POS-MRF becomes

$$p\left(Q,D\right)=\frac{1}{Z}\left\{\prod_{c\in \{F,O,U\}}\prod_{t\in T}\prod_{q_{i}\in t}f{\left(c,q_{i},D\right)}^{\lambda_{t}\theta_{c}}|\}\right\},$$

where c denotes the clique set associated with one of the three dependency types, $\theta_{c}$ is the parameter associated with the clique set c, $f\left(c,q_{i},D\right)$ is the potential function associated with the query term $q_{i}$ in the clique set c and Z is the normalization function. Taking the logarithm of both sides of the above joint probability function and applying Bayes’ rule, we can get the probability of retrieving document D given query Q:

$$\log p(D|Q)=\sum_{\in \{F,O,U\}}\theta_{c}\sum_{t\in T}\lambda_{t}\sum_{q_{i}\in t}\log f(c,q_{i},D)-\log Z-\log p\left(D\right).$$

Since log⁡Z and $\log p\left(D\right)$ do not influence the document ranking, we can define the ranking function as

$$r\left(Q,D\right)=\sum_{\in \{F,O,U\}}\theta_{c}\sum_{t\in T}\lambda_{t}\sum_{q_{i}\in t}\log f(c,q_{i},D).$$

In our system, we utilized heuristics and set $\theta_{c}$ to 0.8, 0.1, 0.1 for dependency types *F*, *O* and *U*, respectively. We utilized the optimal $\lambda_{t}$ which maximized the mean average precision (MAP) based on the TREC 2011 and 2012 Medical Records datasets. The optimal values are 0.5970, 0.2265, 0.3065, 0.2260, 0.3730, 0.1040, 0.8930 and 0.0 for nouns, plural nouns, past participle verbs, past tense verbs, adjectives, adverbs, singular proper nouns and all other POS categories, respectively.

### Medical entity extraction

We extracted the medical entities from both queries and documents. We used an ensemble of the state-of-the-art named entity normalization tools, PubTator (42) and beCAS (43), supplemented by a dictionary-based lookup for identifying the entities and normalizing them to standard identifiers.

First, we used the REST-API services provided by PubTator and beCAS to detect entities from the texts. Subsequently, we built a dictionary by compiling different dictionaries from multiple knowledge sources such as Entrez (44), UniProtKB (45), Gene ontology (46), CTD (47) and MeSH (39), and looked up gene, biological processes, cell component, chemical names and disease names in the composite dictionary. This dictionary lookup resolved three problems where PubTator and beCAS failed: (1) noun phrases lacking morphological features were detected (for example, PubTator and beCAS failed to detect ‘bone morphogenetic protein-2’ while the tokenization component in the dictionary lookup translated the phrase to ‘bone morphogenetic protein 2’ that could be exactly matched in the dictionary Entrez); (2) acronyms were detected and (3) strings with high surface similarity were detected [for example, both ‘Gialpha(1)’ and ‘Gi alpha(2)’ were detected by the dictionary lookup while PubTator failed to detect ‘Gi alpha(2)’].

We had certain priority rules to resolve conflicts between the entity recognition systems. Specifically, we utilized the annotations of PubTator for genes/proteins, chemical and disease names when conflicts existed between PubTator and other systems. When PubTator failed to detect those entities, we considered beCAS and the dictionary lookup. Moreover, when a phrase was matched in more than one dictionary in the dictionary lookup, we chose the dictionary that exactly matched the phrase instead of those with partial matches. More details can be found in the BELMiner toolkit paper (48).

### Query expansion with word embeddings

We utilized the skip-gram word2vec model to generate word embeddings. Suppose a word $w\in V_{w}$ and a context word $c\in V_{c}$ are used as input where $V_{w}$ and $V_{c}$ denote word and context vocabulary in the corpus, respectively. The corresponding embedding vectors are $w\in R^{d}$ and $c\in R^{d}$ where d is the dimension of the embedding vectors. The goal of word2vec is to predict the context words when given a word, i.e. $p\left(c|w\right)=e^{w'c}/\sum_{c\in V_{c}}e^{w'c}$. The embedding vectors could be learned by maximizing the log-likelihood on the training data. However, the intractability of computing $\sum_{c\in V_{c}}e^{w'c}$, Mikolov *et al*. (49) suggests maximizing the following objective likelihood function $\log\sigma (w'c)+kE_{c∼P_{D}}\left(\log\sigma \left(-w^{'}c\right)\right),$ where σ(·) is the sigmoid function, $P_{D}$ is a probability measure on the words to sample false context words and k is the number of false context words for each w. The embedding vectors are then learned by maximizing the revised likelihood function.

Since local embeddings that capture the nuances of topic-specific language perform better than global embeddings, and the latter usually under-perform the former for IR tasks. Therefore, we trained the skip-gram model on the given document collection, i.e. a collection of the ‘title’ and ‘description’ of all the datasets. One hidden layer was utilized and the dimension is set to 100 in the skip-gram model. Minor preprocessing was conducted for the corpus before training, including lowercasing and removing punctuation. Then the entire corpus was merged into one text document to train the word2vec model. We utilized the extracted medical entity terms described in the previous section for expansion. For each medical term, we calculated the cosine similarity in the embeddings and used the five nearest terms as the expansion.

For example, take the query ‘Find data of all types on synaptic growth and remodeling related to glycolysis in the human brain across all databases.’ We first extracted medical entity terms ‘growth,’ ‘glycolysis,’ ‘human,’ ‘brain’ and found the five nearest terms in the embeddings for each term, i.e. ‘factor-i pressure lymphangiogenic factor-a factor-b’ for ‘growth,’ ‘glycolytic phenylpropanoid tca catabolism gluconeogenesis’ for ‘glycolysis,’ ‘murine mutz- mouse mutamouse tert-immortalized’ for ‘human’ and ‘subcortical brainstem thalamic cortical neurochemistry’ for ‘brain.’ As implied in the previous studies (28, 40, 50), low weights were usually given to the expanded query terms while high weights were given to the original query terms. Thus, in our system, we heuristically set the weight for the expanded query terms to 0.1 and original query terms to 0.9. In the previous example, the expanded query, i.e. ‘factor-i pressure lymphangiogenic factor-a factor-b glycolytic phenylpropanoid tca catabolism gluconeogenesis murine mutz- mouse mutamouse tert-immortalized subcortical brainstem thalamic cortical neurochemistry,’ was weighed 0.1 while the original query, i.e. ‘Find data of all types on synaptic growth and remodeling related to glycolysis in the human brain across all data-bases,’ was weighed 0.9. Note that we removed the stopwords from the original query in the retrieval system and added two words ‘find’ and ‘search’ into the stopword list for this specific challenge.

### Re-ranking

Using the afore-mentioned retrieval models and query expansions, we retrieved the top 10 000 datasets for each query. Each document D was associated with a ranking score $s_{D}$ and the highest ranking score (i.e. the ranking score of the document ranked at the first place) was denoted as $s_{max}.$ Then, we re-ranked the retrieved document D based on the number of entities $n_{D}$ that the document had in common with the query. In other words, we conducted an exact match between entities in the queries and those in the documents. We counted the number of shared unique entities between each retrieved document and the query. Using those numbers, we re-calculated the score of each retrieved document and ranked them again and returned the top 1000 documents. We used the following formula to calculate the final score of document D*:*

$$s_{D}^{'}=s_{max}*n_{D}+s_{D}.$$

By doing this, we can assign larger weights to the documents that have more shared entities associated with a query.

## Experiments

In this section, we describe the dataset provided by the BioCADDIE Dataset Retrieval Challenge and present the empirical results of 12 systems based on different settings, including five official participant systems in the challenge and seven comparative systems. These systems were measured by the official metrics and one additional metric. An error analysis is then provided for illustrating the pros and cons of the proposed system.

### Dataset

The organizers generated the dataset collection from DataMed (https://datamed.org/), which was a prototype biomedical data search engine that contains numerous biomedical datasets from a variety of data repositories. The provided dataset collection was derived from a set of 23 individual repositories, which resulted in a total of 794 992 datasets (51). Multiple metadata, including structured, unstructured and semi-structured metadata, were given in the dataset collection, such as ‘title,’ ‘description,’ ‘platform,’ ‘repository’ and ‘species.’ Six queries with retrieved results for which the relevance judgments have been annotated were provided as training data and 15 queries were given as testing data.

A dataset was judged as relevant if it captured all required concepts in the query and if it answered the query or there was a relationship between terms or key concepts. If each key term existed in the dataset title or description, but there was no relationship between terms, the dataset was marked as partially relevant. If no related terms or concepts exist, or the majority of the concepts are missing, the dataset is judged as not relevant. Table 1 shows an example of a query and the relevant and partially relevant dataset. Though there are vast amounts of meta-data available, we observed that the annotation guidelines provided by the organizers implied that the human experts to a great extent annotated the dataset based on the free text in the ‘title’ and ‘description’ fields (see the annotation guidelines at https://github.com/yanshanwang/biocaddie2016mayodata/blob/master/AnnotationGuidelineFinal.pdf). The released six queries with relevance judgments confirmed our observation (see https://github.com/yanshanwang/biocaddie2016mayodata/blob/master/Example_with_Annotation_Qrels_100716_updated.zip). Bouadjenek and Verspoor’s study (53) also shows that querying the ‘title’ and ‘description’ fields provides the best retrieval performance since these two fields are the most common across the repositories. Therefore, in order to mimic human experts’ judgment, we only utilized the unstructured texts in ‘title’ and ‘description’ in our submissions.

**Table 1.** An example of a query and the corresponding relevant and partially relevant datasets

*Query*	Find data on T-cell homeostasis related to multiple sclerosis across all databases
*Relevant dataset*	*Partially relevant dataset*
**Title**: A Combination Trial of Copaxone Plus Estriol in Relapsing Remitting Multiple Sclerosis.	**Title**: Quorum sensing in CD4+ T cells homeostasis: IL-2 coordinates the interplay between IL-2p and regulatory T cells.
**Description**: Through their functional diversification, CD4+ T cells play key roles in both driving and constraining immune-mediated pathology. … Polymorphisms within the locus encoding a transcription factor BACH2 are associated with di-verse immune-mediated diseases including asthma2, multiple sclerosis3, Crohns disease4-5, coeliac disease6, vitiligo7 and type 1 diabetes8. A role for Bach2 in maintaining immune homeostasis, however, has not been established. Here, we define Bach2 as a broad regulator of immune activation that stabilizes im- munoregulatory capacity while repressing the differentiation programmes of mul- tiple effector lineages in CD4+ T cells. Bach2 was required for efficient forma- tion of regulatory (Treg) cells and consequently for suppression of lethal inflam- mation in a manner that was Treg cell dependent. Assessment of the genome- wide function of Bach2, however, revealed that it represses genes associated with effector cell differentiation. Consequently, its absence during Treg polarization resulted in inappropriate diversion to effector lineages. …	**Description**: Many species of bacteria use quorum sensing to sense the amounts of secreted metabolites and adapt their growth according to their population den- sity. We asked whether similar mechanisms would operate in lymphocyte home- ostasis. We investigated the regulation of the size of Interleukin-2-producing CD4+ T-cell (IL-2p) pool using different IL-2-reporter mice. We found that in the absence of either IL-2 or regulatory CD4+ T-cells (Treg) the number of IL-2p-cells increases. Administration of IL-2 decreases the number of cells of the IL-2p-cell subset and pertinently, abrogates their ability to produce IL-2 upon *in vivo* cognate stimulation, while increasing Treg-cell numbers. We propose that control of the IL-2p-cell numbers occurs via a quorum-sensing-like feedback loop where the produced IL-2 is sensed by both the activated CD4+ T-cell pool and by Treg-cells, which reciprocally regulate cells of the IL-2p-cell subset. In conclusion, IL-2 acts as a self-regulatory circuit integrating the homeostasis of activated and regulatory T cells as CD4+ T-cells restrain their growth by monitoring IL-2 levels thereby preventing uncontrolled responses and autoimmunity. Overall design: 2 populations of conventional CD4+ T cell are analysed. 5 replicates for each. GFP- is the control one.
Judgment rationale*: It doesn’t directly mention anything about T-cell homeostasis but Bach 2 is involved in regulation of level of Treg (Which is regulatory T-cells). Also, it mentions the role of Bach 2 in multiple diseases as highlighted.	Judgment rationale*: It talks about Multiple Sclerosis but doesn’t have anything related to T-cell homeostasis.

* Provided by the challenge organizers

### Baseline and evaluation

In this empirical experiment, we evaluated 12 systems based on different settings of the proposed methods. Table 2 lists the setting for each system. TFIDF (official run1), MRF (official run2) and POS-MRF (official run3) were three baseline systems that utilized the conventional tf-idf weighted VSM, MRF and POS-MRF as the retrieval models respectively. TFIDF + WE, MRF + WE and POS-MRF + WE (official run4) added the query expansion using word embeddings in each model. TFIDF + RR, MRF + RR and POS-MRF + RR added the re-ranking step after retrieving the documents. TFIDF + WE + RR, MRF + WE + RR and POS-MRF + WE + RR leveraged both query expansion and re-ranking in the retrieval models. TFIDF, MRF, POS-MRF, POS-MRF + WE and POS-MRF + WE + RR are the five official systems submitted to the BioCADDIE challenge. By comparing these systems, we were able to know the impact of each component on the retrieval system.

**Table 2.** Settings for the evaluated systems

	TFIDF	MRF	POS-MRF	Word Embeddings	Re-ranking
TFIDF (official run1)	•				
TFIDF+WE	•			•	
TFIDF+RR	•				•
TFIDF+WE+RR	•			•	•
MRF (official run2)		•			
MRF+WE		•		•	
MRF+RR		•			•
MRF+WE+RR		•		•	•
POS-MRF (official run3)			•		
POS-MRF+WE (official run4)			•	•	
POS-MRF+RR			•		•
POS-MRF+WE+RR (official run5)			•	•	•

Five metrics, including inference Average Precision (infAP) (52), inference normalized Discounted Cumulative Gain (infNDCG) (52), NDCG@10 (NDCG at the top 10 documents), P@10(+partial) (precision at the top 10 document including partially relevant datasets) and P@10(-partial) (precision at the top 10 document excluding partially relevant datasets), were used by the challenge organizers to measure the submitted systems. We also computed the MAP as an additional metric. The evaluation scripts for computing these metrics are available at: https://github.com/yanshanwang/biocaddie2016mayodata.

### Preprocessing and indexing

Minor preprocessing was conducted for the corpus, including lowercasing and stopwords removal. Two document types, namely json and xml, were provided in this shared task. We used the json format to extract the title and description fields to construct documents. After the preprocessing, we built an index using Elasticsearch (https://www.elastic.co/), which is an open source package for indexing and retrieving documents. Compared to other IR tools, Elasticsearch is much faster for indexing and searching. It has been adopted by many commercial companies, such as eBay, Dell and Facebook, to handle all kinds of search functionalities. We indexed the ‘title’ and ‘description’ into two fields in Elastic search and utilized both fields simultaneously for retrieval.

## Results

Table 3 lists three examples of the original queries, and the associated extracted medical entities from the original queries and expanded query terms using word embeddings. We can see that the medical entity extraction method successfully extracted the medical entities that were the key medical concepts to understand the query. Since the identical method was applied to the corpus, these medical entities in each document could also be extracted. In the re-ranking step, the exact matching between the medical entities from the query and corpus could dramatically increase the ranking of the relevant datasets. We can also observe that expanded query terms using word embeddings added semantically related terms to the original query. For example, ‘phenylpropanoid’ and ‘gluconeogenesis’ are related to ‘glycolysis’; and ‘progesterone’ and ‘hormone’ are related to ‘estrogen’ for ‘women.’ Adding these related terms could increase the retrieval of relevant or partially relevant datasets. In our system, we assigned a lower weight (weight = 0.1) to the expanded query terms because we wanted the IR system to focus more on the original query terms. By doing so, we could not only reduce the impact of noisy information but also take advantage of the related terms.

**Table 3.** Examples of original queries, extracted medical entities from the original queries and expanded query terms using word embeddings

Original query	Extracted medical entity			Expanded query terms	
	Entity ID	Semantic type	Entity	Entity term	Expanded terms
Find data of all types on synaptic growth and remodeling related to glycolysis in the human brain across all databases.	T0	PROC	Growth	Growth Glycolysis Human Brain	Factor-i pressure lymphangiogenic factor-a factor-b Glycolytic phenylpropanoid tca catabolism gluconeogenesis Murine mutz- mouse mutamouse tertimmortalized Subcortical brainstem thalamic cortical neurochemistry
	T1	PROC	Glycolysis		
	T2	SPEC	Human		
	T3	ANAT	Brain		
Search for data on BRCA gene mutations and the estrogen signaling pathway in women with stage I breast cancer.	T0	PROC	Gene mutations	Gene Mutations Estrogen Signaling Pathway Women Stage Breast Cancer	Expression differential microrna mirna profiles Mutation truncating mutated deletions missense Oestrogen progesterone androgen hormone progestins Signaling autophagy jakstat jak-stat endocytosis Signaling jakstat wnt\ub-catenin signaling nf-kb Men premenopausal pre-menopausal desiring perimenopausal ii-iii uicc iiiiv iiiciv iiic Prostate colorectal ovarian er+ cancers Prostate castrate-resistant breast non-metastatic colorectal
	T1	PATH	Estrogen signaling pathway		
	T2	CHED	Estrogen		
	T3	PROC	Signaling pathway		
	T4	SPEC	Women		
	T5	DISO	Stage I breast cancer		

Table 4 shows the experimental results using the official evaluation scripts in terms of infAP, infNDCG, NDCG@10, P@10(+partial), P@10(-partial) and MAP.

**Table 4.** Experimental results on the BioCADDIE dataset

	infAP	infNDCG	NDCG@10	P@10(+partial)	P@10(-partial)	MAP
TFIDF (official run1)	0.1393	0.3485	0.5735	0.7267	0.2600	0.1708
TFIDF+WE	0.1392	0.3470	0.5735	0.7267	**0.2667**	0.1708
TFIDF+RR	0.1399	0.3404	0.5345	0.6933	**0.2667**	0.1476
TFIDF+WE+RR	0.1484	0.3358	0.5418	0.7067	0.2467	0.1633
MRF (official run2)	0.1424	0.3516	0.5726	**0.7467**	0.2533	**0.1742**
MRF+WE	0.1424	0.3508	**0.5901**	**0.7467**	0.2533	0.1741
MRF+RR	0.1383	0.3439	0.5267	0.6933	0.2467	0.1463
MRF+WE+RR	0.1499	0.3381	0.5564	0.7267	0.2467	0.1659
POS-MRF (official run3)	0.1077	0.3006	0.4406	0.5333	0.2267	0.1273
POS-MRF+WE (official run4)	0.1423	0.3253	0.4453	0.5400	0.2333	0.1640
POS-MRF+RR	0.1382	0.3641	0.5105	0.6533	0.2533	0.1472
POS-MRF+WE+RR (official run5)	**0.1628**	**0.3933**	0.5243	0.6667	0.2600	0.1697

Best performance for each metric is highlighted in bold.

First, we observe that the POS-MRF model is inferior to TFIDF and MRF models. The reason is that the POS parser, a crucial part of the POS-MRF model (20), does not perform well on the given queries since these queries are not complete sentences. For example, a testing query is ‘Search for data of all types related to energy metabolism in obese M. musculus’ and the corresponding POS tagging result is ‘Search/NN for/IN data/NNS of/IN all/DT types/NNS related/VBN to energy/NN metabolism/NN in/IN obese/JJ M./NNP musculus/NNS’. 9 out of 13 terms are one of ‘NN,’ ‘NNS,’ ‘VBN,’ ‘JJ’ and ‘NNP’ that are assigned greater weights according to the POS-MRF. Moreover, ‘M.’ is parsed as ‘NNP’ and ‘search’ is mistakenly parsed as ‘NN,’ which are also weighted larger by the POS-MRF model. Documents containing more terms like ‘search’ or ‘M’ are eventually ranked higher than other documents. Thus, the POS-MRF fails to distinguish important terms from less important terms and parses ‘search’ as ‘NN.’ Future directions for improving the POS-MRF may include assigning different weighs to different terms having the same POS, and searching for multiword expressions like ‘M.musculus’ and training the POS tagger on a biomedical corpus.

Second, we observe that adding the query expansion with word embeddings slightly decreases the performance of TFIDF and MRF in terms of infAP and infNDCG, while it slightly increases the performance of TFIDF in terms of P@10(-partial) and the performance of MRF in terms of NDCG@10. This means that expanding the query using word embeddings adds more relevant terms so that the relevant documents are ranked higher in the retrieval results (i.e. more relevant documents are ranked in the top 10). At the same time, we can also see that the query expansion also incorporates more noisy terms, which leads to more non-relevant documents being retrieved (low infAP and infNDCG). It is interesting that almost no changes are found when P@10(+partial) is used as the metric, which is consistent with the result that the most relevant documents are ranked higher using the query expansion. In addition, we observe that the performance of POS-MRF significantly increases (*P* < 0.01 using Wilcoxon test) with the word embeddings based query expansion in terms of all metrics. This result is consistent with our findings that the expanded terms are important for retrieving relevant documents. Adding these terms into the original query alleviates the impact of POS-MRF on the important query terms.

Third, the TFIDF + RR and the MRF + RR under-perform the TFIDF (or the TFIDF + WE) and the MRF (the MRF + WE), respectively, in terms of almost all of the metrics [except for TFIDF measuring by infAP and P@10(-partial)]. These results show that the re-ranking component does not positively improve the retrieval results for the TFIDF and MRF models. However, the POS-MRF + RR out-performs the POS-MRF in terms of all the metrics. The reason might be that the POS-MRF retrieved more relevant documents in the top 10 000 documents than the other two models (since the re-ranking is performed on the top 10 000 documents) but these relevant documents are ranked very low and the re-ranking could rank these relevant documents high into the top 1000 documents.

Compared to using the query expansion or re-ranking alone, adding both components enhances all of the models (i.e. the TFIDF + WE + RR versus the TFIDF + WE or the TFIDF + RR, the MRF + WE + RR versus the MRF + WE or the MRF + RR and the POS-MRF + WE + RR versus the POS-MRF + WE or the POS-MRF + RR) in terms of infAP. However, when other metrics are used, the performance of using both components is superior to that of using only re-ranking but inferior to that of using only query expansion. This is clearly shown by comparing the MAP results of each model. For example, the MAP of TFIDF + WE + RR is 0.1633, which is between that of TFIDF + RR (0.1476) and that of TFIDF + RR (0.1708) and the MAP of MRF + WE + RR is 0.1659, which is between that of MRF + RR (0.1463) and that of MRF + WE (0.1741). This result is consistent with the above findings of the influence of re-ranking. However, the POS-MRF + WE + RR performs better than either the POS-MRF + WE or the POS-MRF-RR. This result shows that the POS-MRF model could take advantage of both the query expansion and the re-ranking.

Finally, the POS-MRF + WE + RR has the best performance among the evaluated methods in terms of infAP and infNDCG, and competitive performance in terms of other metrics. The results indicate that the proposed system performs well overall. The MRF + WE model has the best NDCG@10 and P@10(+partial) and a competitive P@10(-partial). Therefore, it should be considered when only the top 10 retrieved documents are considered. It is also interesting that the simple TFIDF performs well in terms of NDCG@10, P@10(+partial) and P@10(-partial). The TFIDF is a keyword matching approach, which ranks highly the documents containing more matched query terms in the retrieval list. Particularly in the case of dataset retrieval, documents exactly matching the terminologies in a query are obviously judged as relevant according to the relevance judgment guideline. Therefore, when only the first 10 documents are considered, a simple keyword matching approach, such as TFIDF, usually has good performance.

## Conclusion and discussion

In this article, we propose an IR system for biomedical dataset retrieval. The proposed system combines the state-of-the-art retrieval models and leverages the medical entity extraction method, the query expansion based on word embeddings and the re-ranking to enhance the biomedical dataset retrieval. We compared 12 approaches including our participation in the bioCADDIE Dataset Retrieval Challenge in the experiments. Overall, the proposed approach POS-MRF + WE + RR outperforms other approaches in terms of infAP and infNDCG. The MRF + WE model should be considered when only the top 10 retrieved documents are considered. In addition, we showed the impacts of query expansion and re-ranking on the retrieval performance for each approach.

There are two typical cases in which the proposed approach may fail: (1) if there are no shared keywords or medical entities between a query and a relevant dataset, and the query expansion using word embeddings fails to find the relevant terms in the dataset, the proposed system will fail to retrieve the dataset; and (2) when the query contains inclusion or/and exclusion criteria, it is difficult for the proposed IR system to filter out the datasets that do not meet the criteria. Table 5 illustrates two examples for both cases. In Example 1, ‘*Escherichia coli*’ is a specific bacteria that has bacterial ‘chemoraxis,’ thus the dataset is related to the query. Since the query does not contain ‘*Escherichia coli*’ and the query expansion using wording embeddings fails to find ‘*Escherichia coli*,’ the proposed system fails to retrieve this relevant dataset. In Example 2, the dataset is judged non-relevant to the query since the query is to ‘find data on Nuclear Factor-κB (NF-κB)’ in ‘Myasthenia gravis (MG) patients’ where ‘MG patients’ is the criteria for ‘NF-κB.’ However, due to the shared entities ‘NF-κB’ and ‘signaling pathway,’ the dataset was retrieved and ranked at the third position by the proposed system.

**Table 5.** Examples for error analysis

Example 1: False negatives. *Query*: Find protein sequencing data related to **bacterial chemotaxis** across all databases. *Dataset title*: ***Escherichia coli*** 6.0172: ***Escherichia coli*** 6.0172 genome sequencing project. *Dataset description:* N/A
Example 2: False positives. *Query*: Find data on the **NF-κB signaling pathway** in **MG patients**. *Dataset title*: Ginger and its component ameliorated trinitrobenzene sulfonic acid-induced colitis in mice via modulation of NF-κB activity and interleukin-1β (IL-1β) **signaling pathway**. *Dataset description*: Colitis is the common pathological lesion of inflammatory bowel diseases, the major chronic inflammatory diseases of intestinal tracts in humans. In this study, we investigated the therapeutic effects of ginger extract and its component zingerone in mice with 2, 4, 6-trinitrobenzene sulfonic acid (TNBS)-induced colitis. Mice were administered with TNBS and/or various amounts of ginger and zingerone by an intrarectal route. The severity of colitis was evaluated by colonic weight/length ratio, macroscopic lesion, and histological examination. The mechanisms of ginger and zingerone were further elucidated by DNA microarray, ex vivo imaging, and immunohistochemical staining. Our data showed that treatment with ginger extract and zingerone ameliorated TNBS-induced colonic inflammation and injury in a dose-dependent manner. Pathway analysis of ginger- and zingerone-regulated gene expression profiles showed that ginger and zingerone significantly regulated cytokine-related pathways. Network analysis showed that **NF-κB** and IL-1β were key molecules involved in the expression of ginger- and zingerone-affected genes. Ex vivo imaging and immunohistochemical staining further verified that ginger and zingerone suppressed TNBS-induced **NF-κB** activation and decreased the **NF-κB** and IL-1β protein levels in the colon. In conclusion, our data showed that ginger improved the TNBS-induced colitis in mice via modulation of **NF-κB** activity and IL-1β **signaling pathway**. Moreover, zingerone might be the active component of ginger responsible for the amelioration of colitis induced by TNBS. Overall design: A total of 24 mice was randomly divided into four groups of six mice: mock, mice were given with 0.1 ml of 50% ethanol; TNBS, mice were given with 250 mg/kg TNBS in 0.1 ml of 50% ethanol; TNBS/ginger, mice were administered with mixtures containing 250 mg/kg TNBS and various amounts of ginger extract in 0.1 ml of 50% ethanol; TNBS/zingerone, mice were given with mixtures containing 250 mg/kg TNBS and various amounts of zingerone in 0.1 ml of 50% ethanol. Mice were sacrificed 7 days later for histochemical staining, RNA extraction, and ex vivo imaging.

Keywords for relevance judgment are highlighted in bold.

Based the error analysis above, there are a few future directions to improve the proposed system. First, we would like to develop more sophisticated approaches for query expansion using deep learning models. For example, we could use external resources to train word embeddings for query expansion. Though some studies show the local word embeddings are superior to global embeddings, Example 1 in our error analysis indicates that global word embeddings might find the related terms that cannot be found using the local word embeddings. Moreover, we can also take advantage of the semantic types for each extracted term and assign different weights to the expanded terms computed from word embeddings, similar to the approach proposed in Want *et al*.’s study (54). Second, we want to investigate how to take into account inclusion and exclusion criteria in the system. As shown in Example 2, in our error analysis, queries might have criteria that are crucial to exclude some non-relevant retrieved datasets. By doing so, false positives could be reduced in the final retrieved datasets.

One limitation of this study is that the semi-structured and structured metadata provided in the dataset were not utilized for retrieval in the submitted systems. Scerri *et al*. (55) leveraged the semi-structured data to build entity dictionaries to match the user query, and achieved high retrieval performance. Bouadjenek and Verspoor (53) explicitly show that incorporating semi-structured metadata into retrieval mostly decreases the performance. However, they also show that using the metadata in the ‘gene’ field significantly improves the retrieval performance. Therefore, in our future study, we would like to investigate how to leverage the semi-structured and structured metadata in a dataset retrieval system.

We find that the metrics used to evaluate the systems make the comparison difficult. Though different metrics indicate different aspects of an IR system, we observe that one might conclude differently when different metrics are used. For example, we see that the trend of MAP is consistent with that of infNDCG but mostly inconsistent with that of infAP. Another example is that TFIDF + RR out-performs TFIDF in terms of infAP but under-performs TFIDF in terms of infNDCG and MAP. The results of using the metrics considering only the top 10 documents [i.e. NDCG@10 and P@10(+partial)] are usually consistent, as shown in Table 4. Therefore, an IR system should be evaluated by different metrics to explicitly demonstrate the advantages and disadvantages. Moreover, novel metrics should be studied to measure IR systems, particularly for the IR system designed for specific tasks, such as the dataset retrieval task.

## Funding

This work has been supported by the National Institutes of Health (NIH) grants R01LM011934 and R01GM102282. The bioCADDIE Dataset Retrieval Challenge was supported by the NIH grant U24AI117966.

*Conflict of interest*. None declared.
